# Fabrication and Evaluation of Silk Sericin-Derived Hydrogel for the Release of the Model Drug Berberine

**DOI:** 10.3390/gels7010023

**Published:** 2021-02-20

**Authors:** Chi Yan, Jianwei Liang, Hao Fang, Xizhi Meng, Jiale Chen, Zhi Zhong, Qin Liu, Hongmei Hu, Xiaoning Zhang

**Affiliations:** 1 State Key Laboratory of Silkworm Genome Biology, College of Sericulture, Textile and Biomass Sciences, Southwest University, Chongqing 400715, China; yanchi123@email.swu.edu.cn (C.Y.); liangjw@email.swu.edu.cn (J.L.); fangritian@email.swu.edu.cn (H.F.); mxz9988@email.swu.edu.cn (X.M.); lovxury@email.swu.edu.cn (J.C.); 2 Key Laboratory of Mariculture and Enhancement of Zhejiang Province, Zhejiang Marine Fisheries Research Institute, Zhoushan 316021, China; 2008uky@gmail.com (Z.Z.); XZH239@uky.edu (Q.L.); huhm@zju.edu.cn (H.H.)

**Keywords:** silk sericin, thiol-ene, hydrogel, drug delivery

## Abstract

Silk sericin (SS) produced by *Bombyx mori* is normally discarded as waste in manufacturing processes, which causes environmental pollution. Therefore, investigating the use of silk sericin has economic and environmental benefits. As a three-dimensional structure, the sericin-derived hydrogel was explored in different applications. However, many developed gelation procedures raise concerns regarding safety, cost, and duration of gelation time. In this work, “thiol-ene” click chemistry was used to quickly and controllably prepare an SS-derived hydrogel to resolve these early concerns. Then, berberine was loaded and used as a model for investigating the drug-release profiles of the prepared hydrogel. The experimental results revealed that this hydrogel is eligible for a long-term release of berberine. Throughout the antibacterial experiments, the released berberine maintained its antibacterial activity. Our work expands the application of SS in biomedical industries in an eco-friendly way. Furthermore, the discussed strategy could provide a reference for the subsequent development of SS-derived materials.

## 1. Introduction

Silk fiber produced by *Bombyx mori* consists of fibroin fiber and silk sericin (SS) [1]. SS, contributing 15–30% of the total weight of a cocoon, envelops the fibroin fibers together and acts as the protective layer [2]. Most SS is separated from the fibroin fiber and discarded as waste along with water during the silk reeling process. It is estimated that more than 50,000 tons of SS wastewater are released into the environment in silk processing each year [3]. That SS contaminates environmental water because of its high chemical and biological oxygen demand [4]. Therefore, in addition to its positive effect on the economy, the use of SS would play an important role in the prevention of pollution.

As a natural polymer, silk sericin is biocompatible [5] and biodegradable [6]. It has been explored in different medical applications [7,8,9], especially in the form of hydrogel [10,11,12]. However, SS molecules are difficult to crosslink into a form of hydrogel because of their low molecular weights [13]. Therefore, a variety of techniques have been developed to facilitate the gelation process. Wang et al. reported a sericin hydrogel prepared within 60 s, but glutaraldehyde was used as the crosslinking agent. In addition to the cytotoxicity [14], proteins crosslinked with glutaraldehyde are known to often form substantial precipitation due to polymerization [15]. Olivier et al. presented a method for preparing SS-derived hydrogel through enzymatic crosslinking [16]. Because enzymes are expensive, this method increased the preparation cost. In another work, Park et al. stored an SS aqueous solution at 4 °C for gelation, but the gelation time ranged from 1 day to 10 days [17].

“Thiol-ene” click chemistry is a green and eco-friendly strategy for achieving modification and functionalization of materials. It can crosslink the thiol group and alkene group through photoinitiation quickly and inexpensively [18,19,20,21]. The reaction can proceed through a simple mechanism and is insensitive to the presence of water and oxygen. Therefore, “thiol-ene” click chemistry is easy to scale up under mild conditions [22,23]. In this work, reduced glutathione (GSH), which includes a thiol group, was grafted onto the SS molecule via a carbodiimide coupling reaction. Subsequently, “thiol-ene” click chemistry was used for crosslinking the thiolated SS and poly(ethylene glycol) diacrylate (PEGDA), a biologically inert polymer with a particular use in drug delivery [24], for generating a porous SS-derived hydrogel. This hydrogel exhibits a capability for loading molecular drugs and may be applied as a drug delivery system. Although the preparation of an SS and PEGDA composite hydrogel was first reported by Kunyanee et al. [25], their method requires a 70 min gelation time, and the ammonium persulfate used as the redox agent can irritate the skin and eyes and may cause difficulty in breathing [26].

The aim of this work is to develop a new strategy that can resolve early concerns about SS-derived hydrogels preparation and explore its application as a carrier for drug release, expanding the applications of SS, especially in the biomedical field. As thiol-ene click chemistry is a multifaceted toolbox for small molecule and polymer involved reactions [27], our work could provide a reference for the development of SS-derived material in its many forms.

## 2. Results and Discussion

### 2.1. Gelation Exploration

**Figure 1:**
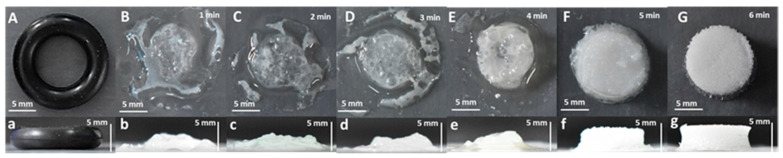
(**A**) is the mold for hydrogel fabrication. (**B**–**G**) representative top view images of a 10% silk sericin (SS)-poly(ethylene glycol) diacrylate (PEGDA) mixture illuminated under a 405 nm LED for 1 min, 2 min, 3 min, 4 min, 5 min, and 6 min, respectively; (**a**–**g**) corresponding side-view images of (**A**–**G**). To generate a stable, well-defined hydrogel, 6 min of 405 nm light-emitting diode (LED) illumination is required.

A 10% SS solution was mixed with PEGDA solution in a volume ratio of 1:1, referred to 10% SS-PEGDA, and then the mixture was illuminated under a 405 nm light-emitting diode (LED), as described in Section Materials and Methods. The duration of LED illumination has a significant influence on the gelation process. Figure 1 suggests that the gelation occurs once the mixtures start receiving illumination from the LED and progress toward a stable, well-defined hydrogel after 6 min of LED illumination. Therefore, 6 min was selected as the appropriate reaction time for the hydrogel scaffold construction (Figure 1).

**Figure 2:**
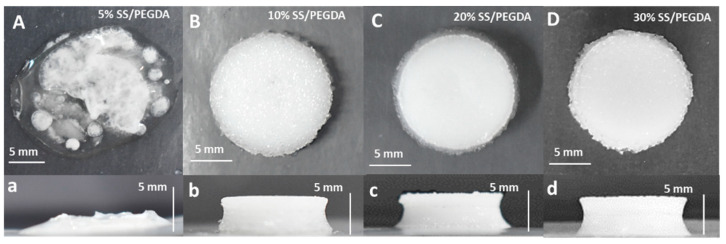
(**A**–**D**) representative top view images of samples 5%, 10%, 20%,and 30% SS/PEGDA; (**a**–**d**) corresponding side-view images of (**A**–**D**).

In addition, the effect of SS solution concentration on hydrogel formation was evaluated. A 5%, 10%, 20% and 30% thiolated SS solution was mixed with PEGDA in a volume ratio of 1:1, denoted 5% SS-PEGDA, 10% SS-PEGDA, 20% SS-PEGDA, and 30% SS-PEGDA, respectively. The samples generated were called 5% SS/PEGDA, 10% SS/PEGDA, 20% SS/PEGDA, and 30% SS/PEGDA, respectively (Figure 2). The results revealed that 5% thiolated SS solution cannot result in a stable, well-defined hydrogel scaffold under the conditions described in Methods, indicating that 5% thiolated silk sericin is not sufficient for crosslinking PEGDA.

### 2.2. Investigation of the Gelation Process

**Figure 3:**
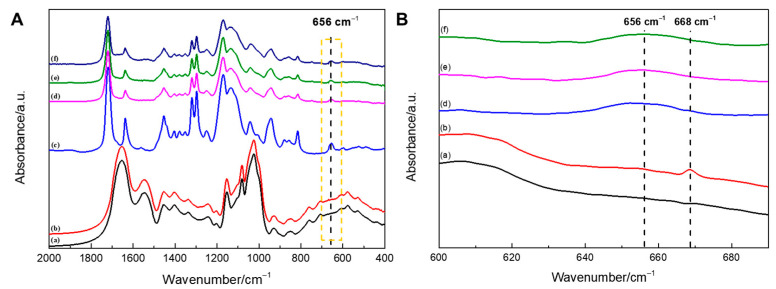
(**A**) Infrared spectra of samples SS (a), thiolated SS/reduced glutathione (GSH)–SS (b), PEGDA (c), 10% SS/PEGDA (d), 20% SS/PEGDA (e), and 30% SS/PEGDA (f); (**B**) enlargement of the area marked by a dashed line in (**A**).

Fourier-transform infrared (FTIR) spectroscopy was used to analyze the differences in functional groups of the samples during preparation. Although a weak peak (Figure 3B) associated with the thiol groups was revealed at 668 cm^−1^ after SS thiolation [28], its change was concealed by the characteristic peak of PEGDA located near 656 cm^−1^ once the thiolated SS was mixed with PEGDA and illuminated under the LED (Figure 3A). Therefore, the changes in the FTIR spectra during the gelation process are difficult to observe in this case.

**Figure 4:**
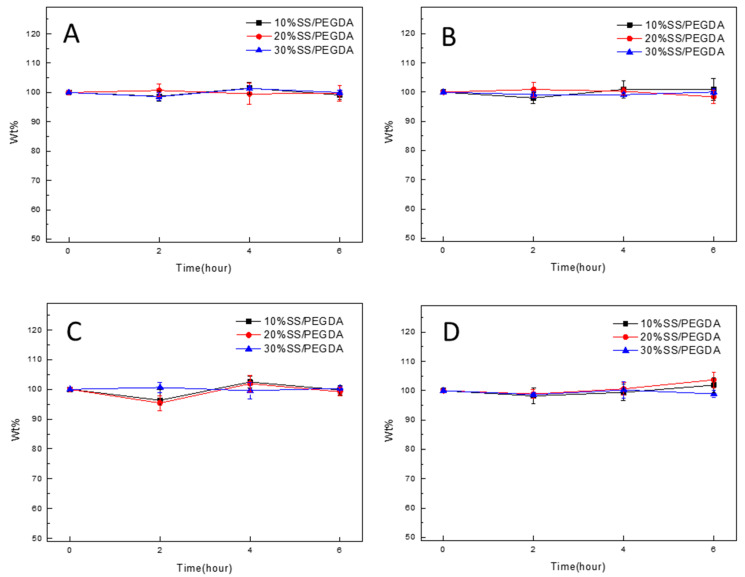
Effects of (**A**) urea, (**B**) NaCl, (**C**) Tween-20, and (**D**) sodium dodecyl sulfate (SDS) on the gelation. Wt % of samples measured at various time intervals in different solutions are not significantly different. The statistical analyses were performed using an unpaired, two-tailed *t*-test.

To better understand the crosslinking of SS and PEGDA molecules within the hydrogel, the prepared samples were immersed in 1 M urea, 1 M NaCl, 1.5% Tween-20, and 0.5% sodium dodecyl sulfate (SDS). Urea was used to disrupt the hydrogen bonds; NaCl was utilized to adjust the ionic strength to disrupt electrostatic interactions; Tween-20 was applied to disrupt hydrophobic interactions; SDS was used as a denaturing detergent to induce protein unfolding via the interruption of intramolecular hydrophobic interactions. The results (Figure 4) show that the weight of the samples in each group remained almost constant over time, indicating that the different non-covalent interactions have a trivial effect on the gelation, and the network is covalently crosslinked.

### 2.3. Microstructural Characterization of SS/PEGDA Scaffolds

**Figure 5:**
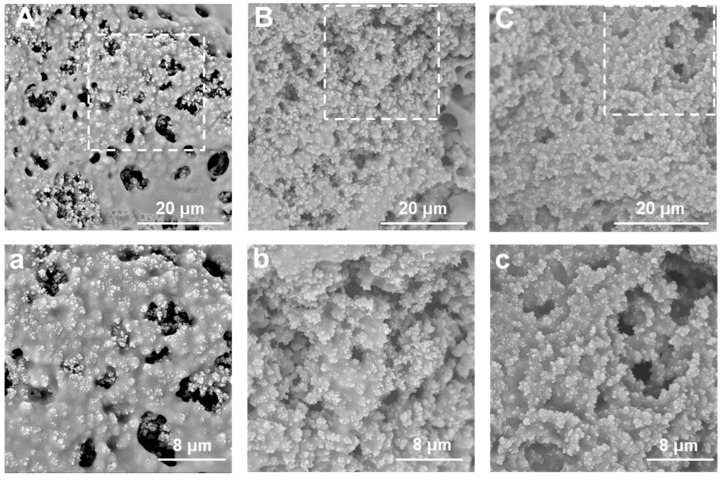
Representative SEM images of samples 10% SS/PEGDA (**A**), 20% SS/PEGDA (**B**), and 30% SS/PEGDA (**C**); (**a**–**c**) are the zoom-in SEM images of areas marked by a dashed line in (**A**–**C**).

The micromorphological structures of the prepared scaffolds are depicted by scanning electron microscopy (SEM) in Figure 5. These SEM images reveal that the porosity of the scaffold structure changed from macroporous to mesoporous with increasing silk sericin content. As the freeze-dried PEGDA hydrogel scaffold (Figure S1) exhibits a pin-hole-free structure under SEM observation (Figure S2), we believe it is the blending of SS that results in a porous structure. On the other hand, the continued addition of SS could lead to a higher crosslinking density; therefore, reduce the pore size and minimize the number of pores, which can be observed in Figure 5C.

Table 1 lists the results of the Brunauer–Emmett–Teller (BET) analysis of the SS/PEGDA scaffolds regarding the specific surface area. Notably, the specific surface area of the scaffolds increased with the increase in SS content from 10% to 20%, then decreased as the SS content increased to 30%. We believe the decreased specific surface area of sample 30% SS/PEGDA is due to the reduced pore size and pore number caused by the increased crosslinking density, which complies with the SEM observations.

### 2.4. Mechanical Behavior of SS/PEGDA Scaffolds

**Figure 6:**
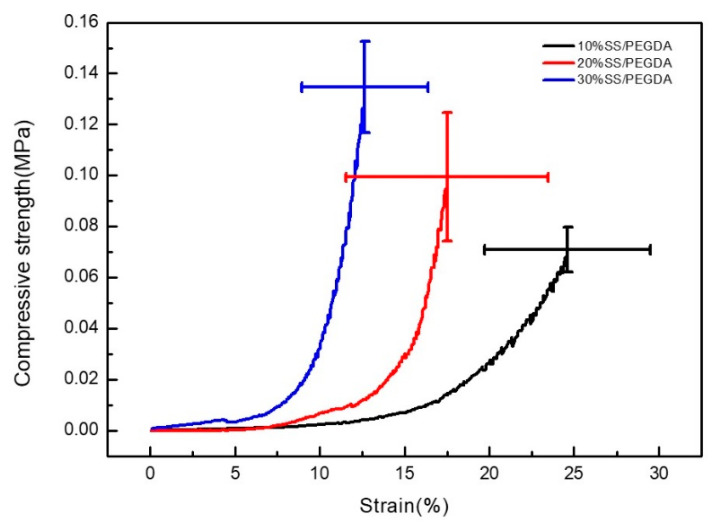
Stress–strain curves on compression test. The mechanical behavior varied significantly between samples 30% SS/PEGDA and 10% SS/PEGDA (*p* < 0.05). The statistical analyses were performed using an unpaired, two-tailed *t*-test.

To extract additional structural properties, the compression test was used to provide indirect information of the scaffold network (Figure 6). The compressive strength of the SS/PEGDA scaffold increased with the increased silk sericin content. The 30% SS/PEGDA scaffolds showed significantly higher compressive strength than the 10% SS/PEGDA scaffold. In contrast, the increased content of silk sericin reduced the strain% of the scaffold. The increased SS content led to a higher crosslinking density, thereby improving the compressive strength of the SS/PEGDA scaffolds. On the other hand, the higher crosslinking density limited the deformation of the SS/PEGDA scaffold under a load and reduced the strain% [29].

Because the 20% SS/PEGDA hydrogel scaffold exhibits the largest specific surface area and appropriate mechanical properties among the samples, it was selected for the follow-up experiments of this study.

### 2.5. Swelling of SS/PEGDA Scaffolds

**Figure 7:**
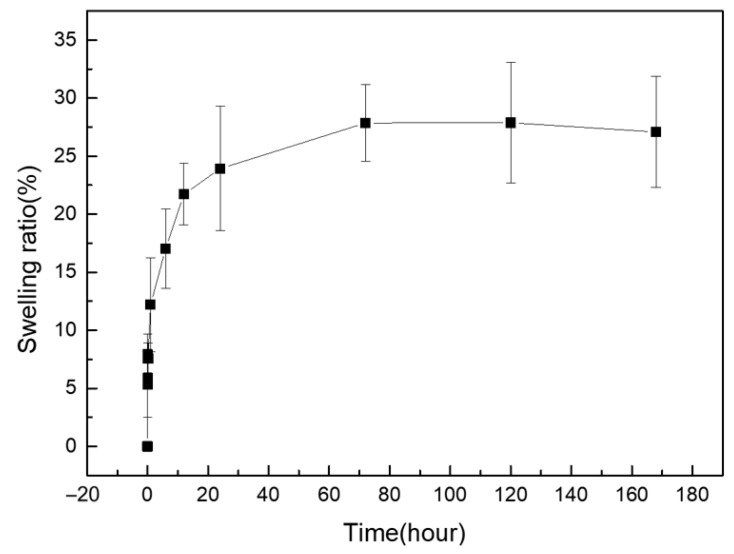
Swelling ratio curve for 20% SS/PEGDA scaffolds in PBS solution. No significant change in the swelling ratio occurs at 72 h, 120 h, and 168 h. The statistical analyses were performed using an unpaired, two-tailed *t*-test.

The swelling ratio has been considered a measurement of the free water within a hydrogel matrix [30]. The lyophilized 20% SS/PEGDA scaffolds underwent swelling instead of dissolution when the samples were placed in PBS buffer. As shown in Figure 7, the swelling ratio of the 20% SS-PEGDA scaffold seemed to reach an equilibrium over 80 h in PBS solution (Figure 7). The swelling behavior indicates that the porous structure within the hydrogels allows for solvent uptake. This swelling property might be useful for drug release.

### 2.6. Kinetics of Drug Release

**Figure 8:**
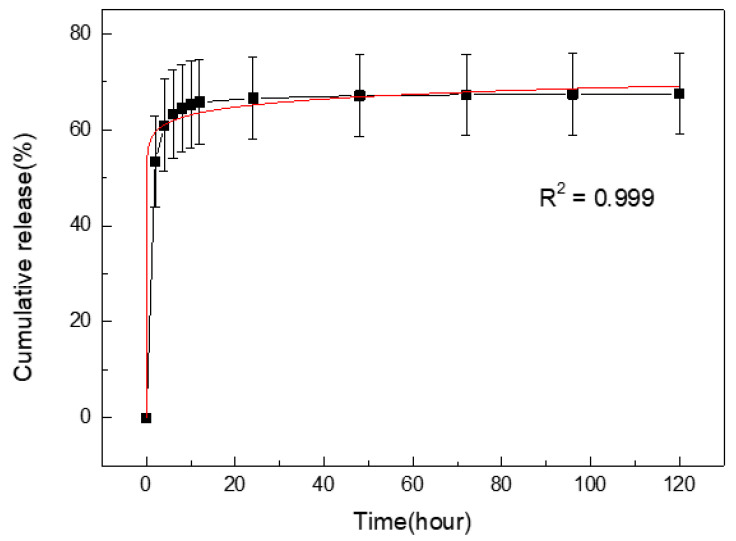
Ritger–Peppas equation fitting (the red line) was applied to the release profile (the black line) using Origin software. The *n* value of the Ritger–Peppas equation is less than 0.45, which indicates that Fickian diffusion is the mechanism of berberine release. No statistically significant differences at 24 h, 48 h, 72 h, 96 h and 120 h were detected. The statistical analyses were performed using an unpaired, two-tailed *t*-test.

Berberine was selected as the model for investigating the practical use of SS/PEGDA in drug delivery, as it has been reported to possess a broad-spectrum antibacterial activity and has anti-inflammatory properties. The kinetics of berberine released from the 20% SS/PEGDA scaffold was examined (Figure 8). The experiment was conducted for a 120 h period, and the release profile demonstrates an initial burst phase followed by a near-zero-order phase (the points after 24 h show a plateau). The initial burst release may result from the rapid release of surface-associated drug molecules [31]. The berberine release data were fit to the Ritger–Peppas equation (Figure 8), and the results indicate the berberine transport mechanism is primarily in agreement with Fickian diffusion [32].

### 2.7. Antibacterial Property

**Figure 9:**
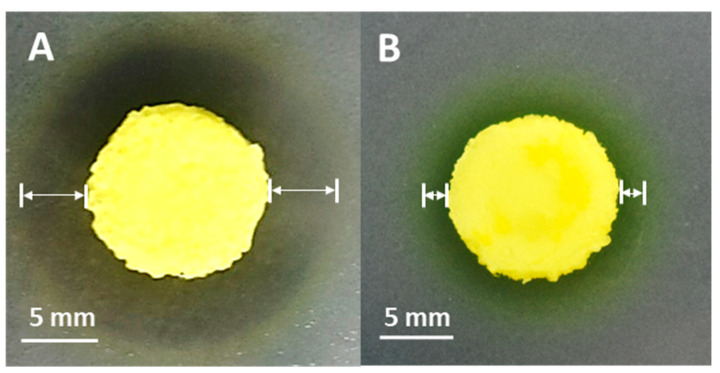
Bacterial zone of inhibition studies (**A**) *S. aureus,* (**B**) *E. coli.* The inhibition zones are indicated by the white arrows.

The antibacterial property of the berberine-loaded SS/PEGDA scaffolds was evaluated via the zone of inhibition using the agar disk diffusion method. The scaffolds produced an extensive zone of inhibition against *Staphylococcus aureus* (*S. aureus*, Figure 9A) but demonstrated a limited zone of inhibition when they were placed on *Escherichia coli* (*E. coli*, Figure 9B) spread agar plates. These results are reasonable, as *S. aureus* is more sensitive to berberine than *E. coli* [33]. The zone of inhibition indicates that the antibacterial agent released from SS/PEGDA scaffolds can prevent the formation of bacterial colonies and then bacterial films on the agar plates. The above results suggest that the prepared SS/PEGDA scaffolds have the potential to be used for drug delivery applications.

### 2.8. In Vitro Cytotoxicity

**Figure 10:**
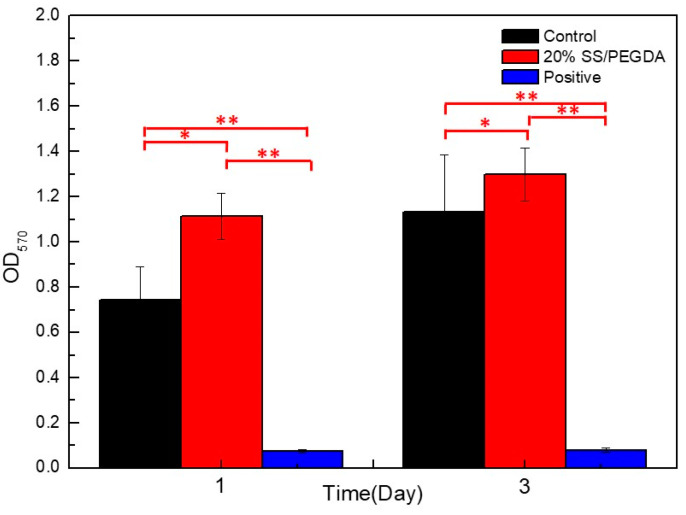
MTT results are expressed as optical density (λ = 570 nm) readings, and are means ± SD, *n* = 3. Statistical analyses were performed using an unpaired, two-tailed *t*-test (* *p* < 0.05, ** *p* < 0.01).

The MTT assay was applied to evaluate the effects of leaching liquor from 20% SS/PEGDA hydrogel scaffolds on the proliferation and viability of human embryonic kidney 293 (HEK-293) cells. The results demonstrate 20% SS/PEGDA hydrogel scaffolds exhibit non-toxicity and have acceptable biocompatibility and potential for clinical use. This conclusion can be drawn from the observation that more viable cells are found in the 20% SS/PEGDA group than in the control group on day 1 and day 3 (Figure 10). The results are consistent with the previous reports that silk sericin can accelerate cell proliferation [34,35]. Representative pictures showing the effects of the leaching liquor on cellular growth can be found in the Supplementary Materials (Figure S3).

## 3. Conclusions

In summary, relying on “thiol-ene” click chemistry, we have developed and designed a strategy for preparing SS-derived hydrogel. Subsequently, the hydrogel scaffold was prepared using a lyophilization procedure and presented a porous structure. Among all sample groups, the 20% SS/PEGDA hydrogel scaffold exhibits a large specific surface area and appropriate mechanical properties. Moreover, results on swelling behavior, drug release, and in vitro cytotoxicity demonstrate that the 20% SS/PEGDA hydrogel scaffold is eligible for drug delivery. Furthermore, our study provides a practical pathway and foundation that could be incorporated into the preparation of SS-derived materials in many forms. It is our hope to promote the application of silk sericin through this developed strategy, achieving social and economic benefits.

## 4. Materials and Methods

### 4.1. Materials

Sericin (99.0%, purified from silk degumming wastewater of the textile industry, Ningshan Guosheng Biological Technology Co. Ltd., Ningshan, China), Tris(2-carboxyethyl) phosphine hydrochloride (TCEP·HCl, 98%, Aladdin Agent Co. Ltd., Shanghai, China), berberine chloride hydrate (98%, Aladdin Agent Co. Ltd., Shanghai, China), 2-morpholinoethanesulfonic acid (MES, 99%, Aladdin Agent Co. Ltd., Shanghai, China), Sodium chloride (≥99.5%, Aladdin Agent Co. Ltd., Shanghai, China), Sodium carbonate (≥99.8%, KeLong Chemical Reagent Co. Ltd., Chengdu, China), Sodium biphosphate dihydrate (≥99.0%, KeLong Chemical Reagent Co. Ltd. Chengdu, China), Sodium dihydrogen phosphate (≥99.0%, Fangzheng reagent Co. Ltd., Tianjin, China), N-hydroxysuccinimide (NHS, 99.0%, Solarbio Co. Ltd., Beijing, China), reduced glutathione (GSH, 98%, Solarbio Co. Ltd., Beijing, China), 1-(3-dimethylaminopropyl)-3-ethylcarbodiimide hydrochloride (EDC, ≥97%, Adamas-beta, Shanghai, China), poly(ethylene glycol) diacrylate (PEGDA, 99.9%, n = approximately 4, Tokyo Chemical Industry Co. Ltd., Tokyo, Japan), potassium bromide (>99.0%, Sangon Biotech Co. Ltd., Shanghai, China), Dulbecco’s modified Eagle’s medium (DMEM, high glucose, HyClone, Logan, UT, USA), phosphate-buffered saline (PBS, 1×, HyClone, Logan, UT, USA), 3-(4,5-dimethythiazol-2-yl)-2,5-diphenyl tetrazolium bromide (MTT, 98%, BioFroxx., Einhausen, Germany), dimethyl sulfoxide (DMSO, 100%, BioFroxx., Einhausen, Germany), fetal bovine serum (FBS, Zhejiang Tianhang Biotechnology Co. Ltd., Huzhou, China).

### 4.2. Silk Sericin Molecule Thiolation

A 30 g of silk sericin was dissolved in 200 mL of deionized water (diH_2_O) and dialyzed against 2-morpholinoethanesulfonic acid monohydrate (MES) solution (containing 0.1 mol/L MES and 0.5 mol/L NaCl) at a pH of 6.0 for 24 h. Then, 1-ethyl-3-(3-dimethylaminopropyl)carbodiimide (EDC) and N-hydroxysuccinimide (NHS) were mixed with the resulting solution for the carboxyl group activation and maintained a final concentration of 0.5 mg/mL and 0.7 mg/mL, respectively, followed by the addition of 10.95 g of GSH. The reaction was conducted at 4 °C for 15 min. The GSH-modified silk sericin (GSH-SS) was dialyzed against diH_2_O for 24 h to remove all reaction residue. The obtained solution was lyophilized (LGJ-10, Shanghai Yuming Automation and Technology Co. Ltd., Shanghai, China) and stored under vacuum for later usage.

### 4.3. Silk Sericin-Derived Hydrogel Scaffold Preparation

Lyophilized GSH-SS powder was dissolved in diH_2_O to prepare 5%, 10%, 20%, and 30% GSH-SS solution. Then, TCEP•HCl was added to the above solutions to obtain the final concentration of 5 mmol/L, 10 mmol/L, 20 mmol/L, and 30 mmol/L, respectively, to break the disulfide bonds possibly formed to release free thiol groups. A 1 mL aliquot of PEGDA solution was then thoroughly mixed with 1 mL of TCEP•HCl-reduced GSH-SS solution and placed 3 cm below a LED light source (405 nm, Shenzhen YuXianDe Science and Technology Ltd., Shenzhen, China) for illumination of various duration. The PEGDA molecules crosslinked thiolated SS molecules to generate SS-derived hydrogels, which were then lyophilized for SS/PEGDA scaffold preparation.

### 4.4. FTIR Analysis

The freeze-dried hydrogel scaffolds were ground to powder, which was mixed with KBr in a mass percentage concentration (*w/w*) of 100:1. The mixture was pressed into a fine disc, which was scanned at a resolution of 4 cm^−1^ over a wavenumber of 400–4000 cm^−1^ for 24 scans by Fourier-transform infrared spectroscopy (Nicolet iN10, Thermo Scientific, Waltham, MA, USA).

### 4.5. Effect of Tween-20, PBS, NaCl and Urea on the Gelation

PBS solutions (pH = 7.4) containing the indicated concentration of Tween-20, SDS, NaCl, and urea were prepared. The weights of the SS/PEGDA hydrogels were measured with an analytical balance and recorded as *m_i_*. Next, the samples were immersed in 6 mL of the above solutions for different durations. At certain time points, the samples were removed from the solutions, and the extra solution on their surface was gently wiped with Kimwipes. Then, the measurement of sample weight was recorded as *m_f_*. The change in weight can be reflected by using the following equation:

(1) $$Wt\;\%\;=\;\frac{m_{f}}{m_{i}}\times 100\%$$

### 4.6. Microstructural Morphology Characterization

The sample was cut through and coated with a thin layer of gold via a sputter coater (GSL-1100×-SPC-16 M, MTI Corporation, Richmond, VA, USA). The morphology and pores of the scaffold were then analyzed using a scanning electron microscope (SEM, Phenom Pro, Phenom-World, Eindhoven, The Netherland).

### 4.7. BET Analysis

The specific surface area of the freeze-dried hydrogel scaffolds was investigated. The analysis was done at 50 °C on a surface area and porosity measurement system (ASAP 2460, Micromeritics Instruments Corporation, Richmon, VA, USA). Additionally, the specific surface area was calculated using the Brunauer–Emmett–Teller (BET) method.

### 4.8. Compression Test of the SS/PEGDA Scaffold

The compression test was performed at a compressive rate of 2 mm/min at 25 °C. The universal testing machine (GTM-2100, Shanghai Xieqiang Instrument Technology Co. Ltd., Shanghai, China) was stopped when the strain of the SS/PEGDA scaffold reached 50%.

### 4.9. Study of Swelling Behavior

Each SS/PEGDA scaffold prepared under different conditions was weighed first, and then the samples were immersed in 6 mL of PBS solution (pH = 7.4) for a different duration. Subsequently, the samples were taken out, and their surface was blotted with a tissue to remove excess solution. The swelling ratio (*SR*) can be determined using the following equation:

(2) $$SR=\frac{\left(W_{t}-W_{d}\right)}{W_{d}}$$

where *W_t_* is the weight of the hydrogel scaffold after immersion in PBS, and *W_d_* is the weight of the sample after lyophilization.

### 4.10. Berberine-Loaded SS/PEGDA Hydrogel Scaffold Preparation

First, berberine was dissolved in diH_2_O to obtain a solution with a concentration of 3 mg/mL. The berberine-loaded SS/PEGDA scaffolds were obtained by placing each prepared SS/PEGDA scaffold in 10 mL of 3 mg/mL berberine solution under vacuum for 90 min. The samples were then freeze-dried for later usage.

The initial amount of berberine loaded in the SS/PEGDA scaffold can be calculated using the following equation:

(3) $$m_{0}=\left(c_{f}v_{f}-c_{i}v_{i}\right)\times M$$

where *c_f_* and *c_i_* are the concentration of berberine in the solution before and after drug loading, respectively, determined using a UV-vis method and calculated in accordance with the standard curve of berberine in diH_2_O (Figure S4). *v_f_* and *v_i_* are the volume of berberine solution before and after drug loading, respectively. *M* represents the molecular weight of berberine.

### 4.11. Mathematical Modeling of Drug Release from SS/PEGDA Scaffolds

To assess the in vitro release kinetics of berberine-loaded SS/PEGDA scaffolds, each sample was placed in 6 mL of PBS (pH = 7.4) solution for 120 h at 37 °C, with a rotary speed of 100 r/min. The cumulative berberine released at different time intervals was determined from its calibration curve (Figure S5) using a UV-vis spectrophotometer (UV1600, Shanghai Jinghua Technology Instrument Co. Ltd., Shanghai, China) at the wavelength of 345 nm according to the following equation:

(4) $$\mathrm{Cumulative}\;\mathrm{release}\;(\%)\;=\;\frac{\sum_{1}^{n}vC_{t}}{m_{0}}\times 100\%$$

where *v* is 6 mL, and *C_t_* is the concentration of berberine in PBS solution at time *t*. *m_0_* represents the initial amount of berberine loaded. Aliquots of 6 mL were withdrawn and replaced with the same volume of fresh PBS solution after each measurement at the indicated time point.

The Ritger–Peppas model, expressed as follows, was used to fit the release profile using OriginPro 8 software(Originlab Corporation, Northampton, MA, USA):

(5) $$ln\left(\frac{m_{t}}{m_{\infty }}\right)=lnk+nlnt$$

where *m_t_* is the amount of berberine released at time *t*, *m_∞_* represents the amount of berberine released at an infinite time, and the value of *m_t_*/*m_∞_* indicates the proportion of berberine released at time *t*. In addition, *k* represents the kinetic constant, and the exponent *n* is indicative of the drug release mechanism.

### 4.12. Test for Antimicrobial Activity of Berberine-Loaded SS/PEGDA Scaffolds

A 400 µL aliquot of *Staphylococcus aureus* (*S. aureus*, ATCC 25923) or *Escherichia coli* (*E. coli*, ATCC 25922) suspension with a specific concentration of 1 × 10^7^ CFU/mL (by adjusting the turbidity of the bacterial suspension to a McFarland standard) was spread over the surface of prepared agar plates. Then, the freeze-dried berberine-loaded SS/PEGDA scaffolds were gently placed on the surfaces of agar plates. The agar plates were cultivated in the dark for 12 h at 37 °C, and the zone of inhibition (ZoI) around each scaffold was recorded by a digital camera and measured with a caliper.

### 4.13. In Vitro Cytotoxicity Assay

First, the SS/PEGDA scaffolds were incubated with PBS solution (pH = 7.4) thrice, 30 min per incubation, to remove unreacted compounds. The scaffolds were then autoclaved at 121 °C for 20 min and dried in an oven at 60 °C. The sterilized samples were subsequently immersed for 72 h in the complete growth medium with a sample weight to complete growth medium volume ratio of 0.02 g/mL. Afterward, the obtained leaching liquor was filtered through a 0.22 µm pore size membrane and used for human embryonic kidney 293 (HEK-293) cell cultivation. HEK-293 cells were seeded into a 96-well plate at a density of 2 × 10^5^ cells/well in 100 µL of filtered leaching liquor, complete growth medium and a 0.64% phenol solution of the complete growth medium, denoted the experimental, control, and positive groups, respectively. HEK-293 cells were obtained from the American Type Culture Collection (ATCC, Manassas, VA, USA).

Afterward, 20 µL of MTT solution (5 mg/mL in PBS) was introduced into each well for 4 h of incubation. The media in the wells were then discarded, followed by the addition of 150 µL of DMSO to dissolve the precipitate. The optical density (OD) value was recorded at 570 nm using a microplate reader (Synergy H1, Biotek, Winooski, VT, USA).

## Figures and Tables

**Table 1:** The specific surface area of the prepared hydrogel scaffolds.

Scheme.	Specific Surface Area (m^2^/g)
10% SS/PEGDA	0.1230
20% SS/PEGDA	0.2811
30% SS/PEGDA	0.1411

## Data Availability

The data presented in this study are available on request from the corresponding author.

## Supplementary Materials

The following are available online at https://www.mdpi.com/2310-2861/7/1/23/s1, Figure S1: Representative images of PEGDA hydrogel, Figure S2: Representative SEM images of the lyophilized PEGDA hydrogel, Figure S3: The morphologies of HEK-293 cells of each group in MTT assay on day 1 and day 3, Figure S4: Standard calibration curve of curcumin in diH2O, Figure S5: Standard calibration curve of curcumin in PBS (pH = 7.4).

## References

[1] Kim U.-J., Park J., Li C., Jin H.-J., Valluzzi R., Kaplan D.L. (2004). Structure and Properties of Silk Hydrogels. Biomacromolecules.

[2] Santos N.T.D.G., Landers R., da Silva M.G.C., Vieira M.G.A. (2020). Adsorption of Gold Ions onto Sericin and Alginate Particles Chemically Crosslinked by Proanthocyanidins: A Complete Fixed-Bed Column Study. Ind. Eng. Chem. Res..

[3] Arango M.C., Montoya Y., Peresin M.S., Bustamante J., Álvarez-López C. (2020). Silk Sericin as A Biomaterial for Tissue Engineering: A Review. Int. J. Polym. Mater. Polym. Biomater..

[4] Kunz R.I., Brancalhão R.M.C., Ribeiro L.D.F.C., Natali M.R.M. (2016). Silkworm Sericin: Properties and Biomedical Applications. BioMed Res. Int..

[5] Mitran V., Dinca V., Ion R., Cojocaru V.D., Neacsu P., Dinu C.Z., Rusen L., Brajnicov S., Bonciu A., Dinescu M. (2018). Graphene Nanoplatelets-Sericin Surface-Modified Gum Alloy for Improved Biological Response. RSC Adv..

[6] Kumar J.P., Mandal B.B. (2017). Antioxidant Potential of Mulberry and Non-mulberry Silk Sericin and its Implications in Biomedicine. Free Radic. Biol. Med..

[7] Lamboni L., Gauthier M., Yang G., Wang Q. (2015). Silk sericin: A Versatile Material for Tissue Engineering and Drug Delivery. Biotechnol. Adv..

[8] Elahi M., Ali S., Tahir H.M., Mushtaq R., Bhatti M.F. (2020). Sericin and Fibroin Nanoparticles—Natural Product for Cancer Therapy: A Comprehensive Review. Int. J. Polym. Mater..

[9] Sunaina S., Subhayan D., Mahitosh M., Ghosh A.K., Kundu S.C. (2018). Prospects of Nonmulberry Silk Protein Sericin-based Nanofibrous Matrices for Wound Healing—In Vitro and In Vivo Investigations. Acta Biomater..

[10] Qi C., Deng Y., Xu L., Yang C., Wang L. (2020). A Sericin/Graphene Oxide Composite Scaffold as A Biomimetic Extracellular Matrix for Structural and Functional Repair of Calvarial Bone. Theranostics.

[11] Tao G., Wang Y.J., Cai R., Chang H.P., Song K., Zuo H., Zhao P., Xia Q.Y., He H.W. (2019). Design and Performance of Sericin/Poly (vinyl alcohol) Hydrogel as A Drug Delivery Carrier for Potential Wound Dressing Application. Mat. Sci. Eng. C.

[12] Qi C., Liu J., Jin Y., Xu L.M., Wang G.B., Wang Z., Wang L. (2018). Photo-Crosslinkable, Injectable Sericin Hydrogel as 3D Biomimetic Extracellular Matrix for Minimally Invasive Repairing Cartilage. Biomaterials.

[13] Liu J., Qi C., Tao K., Zhang J., Zhang J., Xu L., Jiang X., Zhang Y., Huang L., Li Q. (2016). Sericin/Dextran Injectable Hydrogel as An Optically Trackable Drug Delivery System for Malignant Melanoma Treatment. ACS Appl. Mater. Interfaces.

[14] Wang Z., Zhang Y., Zhang J., Huang L., Liu J., Li Y., Zhang G., Kundu S.C., Wang L. (2014). Exploring Natural Silk Protein Sericin for Regenerative Medicine: An Injectable, Photoluminescent, Cell-adhesive 3D Hydrogel. Sci. Rep..

[15] Greg T.H. (2013). Chapter 22-Enzyme Modification and Conjugation. Bioconjugate Techniques.

[16] Leile De Almeida M.D.O.A., Isabel M.B.D.S.S., Ferreira Borges S.C., Pereira Alves P.J. (2018). Silk Sericin-based Hydrgel Methods and Uses Thereof. Portugal Patent.

[17] Chun J.P., Jooyeon R., Chang S.K., Joog W.K., Ick S.K., Do G.B., Um I.C. (2018). Effect of Molecular Weight on the Structure and Mechanical Properties of Silk Sericin Gel, Film, and Sponge. Int. J. Biol. Macromol..

[18] Stichler S., Jungst T., Schamel M., Zilkowski I., Kuhlmann M., Böck T., Blunk T., Teßmar J., Groll J. (2017). Thiol-ene Clickable Poly (glycidol) Hydrogels for Biofabrication. Ann. Biomed. Eng..

[19] Zhang Y., Chu C.W., Ma W., Takahara A. (2020). Functionalization of Metal Surface via Thiol–Ene Click Chemistry: Synthesis, Adsorption Behavior, and Postfunctionalization of a Catechol- and Allyl-Containing Copolymer. ACS Omega.

[20] Ji S.L., Qian H.L., Yang C.X., Zhao X., Yan X.P. (2019). Thiol-Ene Click Synthesis of Phenylboronic Acid-Functionalized Covalent Organic Framework for Selective Catechol Removal from Aqueous Medium. ACS Appl. Mater. Interfaces.

[21] Felgueiras H.P., Wang L.M., Ren K.F., Querido M.M., Jin Q., Barbosa M.A., Ji J., Martins M.C.L. (2017). Octadecyl Chains Immobilized onto Hyaluronic Acid Coatings by Thiol-ene “Click Chemistry” Increase the Surface Antimicrobial Properties and Prevent Platelet Adhesion and Activation to Polyurethane. ACS Appl. Mater. Interfaces.

[22] Hoyle C.E., Bowman C.N. (2010). Thiol-Ene Click Chemistry. Angew. Chem. Int. Edit..

[23] Xu J., Boyer C. (2015). Visible Light Photocatalytic ThiolEne Reaction: An Elegant Approach for Fast Polymer Postfunctionalization and Step-Growth Polymerization. Macromolecules.

[24] Liang J., Zhang X., Chen Z., Li S., Yan C. (2019). Thiol-Ene Click Reaction Initiated Rapid Gelation of PEGDA/Silk Fibroin Hydrogels. Polymers.

[25] Punyamoonwongsa P., Klayya S., Sajomsang W., Kunyanee C., Aueviriyavit S. (2019). Silk Sericin Semi-interpenetrating Network Hydrogels Based on PEG-Diacrylate for Wound Healing Treatment. Int. J. Polym. Sci..

[26] (2018). Ammonium Persulfate.

[27] Hoyle C.E., Lowe A.B., Bowman C.N. (2010). Thiol-click Chemistry: A Multifaceted Toolbox for Small Molecule and Polymer Synthesis. Cheminform.

[28] Socrates G. (2001). Sulphur and Selenium Compounds. Infrared and Raman Characteristic Group Frequencies.

[29] Zhao J., Yu P., Dong S. (2016). The Influence of Crosslink Density on the Failure Behavior in Amorphous Polymers by Molecular Dynamics Simulations. Materials.

[30] Bodenberger N., Kubiczek D., Abrosimova I., Scharm A., Kipper F., Walther P., Rosenau F. (2016). Evaluation of Methods for Pore Generation and Their Influence on Physio-Chemical Properties of a Protein Based Hydrogel. Biotechnol. Rep..

[31] Fu Y., Kao W.J. (2010). Drug Release Kinetics and Transport Mechanisms of Non-degradable and Degradable Polymeric Delivery Systems. Expert Opin. Drug Del..

[32] Ritger P.L., Peppas N.A. (1987). A Simple Equation for Description of Solute Release, I. Fickian and Non-Fickian Release from Non-Swellable Devices in the Form of Slabs, Spheres, Cylinders or Discs. J. Control. Release.

[33] Čerňáková M., Košťálová D. (2002). Antimicrobial Activity of Berberine—A Constituent of Mahonia Aquifolium. Folia Microbiol..

[34] Wang F., Hou K., Chen W., Wang Y., Wang R., Tian C., Xu S., Ji Y., Yang Q., Zhao P. (2020). Transgenic PDGF-BB/Sericin Hydrogel Supports for Cell Proliferation and Osteogenic Differentiation. Biomater. Sci..

[35] Bakhsheshi-Rad H.R., Ismail A.F., Aziz M., Akbari M., Hadisi Z., Omidi M., Chen X. (2020). Development of the PVA/CS Nanofibers Containing Silk Protein Sericin as A Wound Dressing: In Vitro and in Vivo Assessment. Int. J. Biol. Macromol..

