# Supplementary material for: Fabrication and Evaluation of Silk Sericin-Derived Hydrogel for the Release of the Model Drug Berberine
Source: Gels. 2021 Feb 20;7(1):23. doi: 10.3390/gels7010023 (PMC8005982; doi:10.3390/gels7010023)
Supplement: Supplementary file 1 [file gels-07-00023-s001.pdf]

Supplementary Materials

Fabrication and Evaluation of Silk Sericin-Derived Hydrogel for the Release of the Model Drug Berberine

[1] State Key Laboratory of Silkworm Genome Biology, College of Sericulture, Textile and Biomass Sciences, Southwest University, Chongqing 400715, China; yanchi123@email.swu.edu.cn (C.Y.); liangjw@email.swu.edu.cn (J.L.); fangritian@email.swu.edu.cn (H.F.); mxz9988@email.swu.edu.cn (X.M.); lovxury@email.swu.edu.cn (J.C.)
[2] Key Laboratory of Mariculture and Enhancement of Zhejiang Province, Zhejiang Marine Fisheries Research Institute , Zhoushan 316021, China; 2008uky@gmail.com (Z.Z.); XZH239@uky.edu (Q.L.); huhm@zju.edu.cn (H.H.)
* Correspondence: xzhang@swu.edu.cn
† These authors contributed equally to this work.

Table of content:

1. Representative images of PEGDA hydrogel 2

2. Representative SEM images of the lyophilized PEGDA hydrogel 2

3. Morphologies of HEK-293 cells of each group in MTT assay on day 1 and day 3 2

4. Standard calibration curve of berberine in diH_2O 3

5. Standard calibration curve of berberine in PBS 3

1. Representative images of PEGDA hydrogel

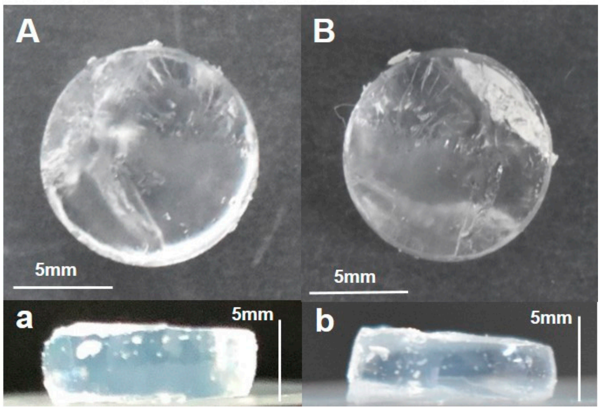

Figure S1. PEGDA hydrogel was prepared by mixing PEGDA and diH_2O in a 1:1 ratio (v/v), then illuminating under a 405 nm LED for 30 minutes. (**A**) and (**B**) are the top view images of the prepared PEGDA hydrogel before and after lyophilization, respectively; (**a**) and (**b**) are the corresponding side view images of (**A**) and (**B**).

2. Representative SEM images of the lyophilized PEGDA hydrogel

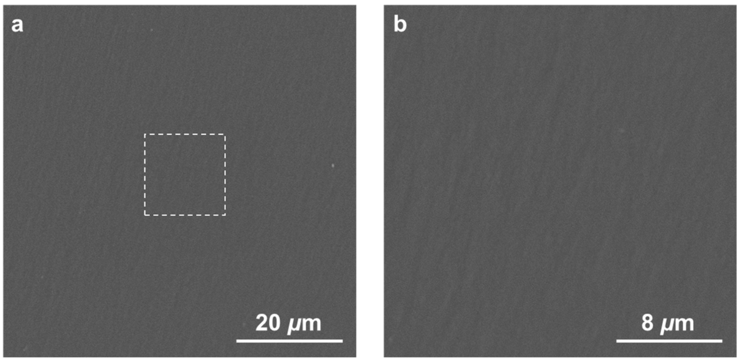

Figure S2. (**a**) shows representative SEM images of the lyophilized PEGDA hydrogel; (**b**) is the zoom-in SEM image of the region marked by a dashed line in (**a**).

3. Morphologies of HEK-293 cells of each group in the MTT assay on day 1 and day 3

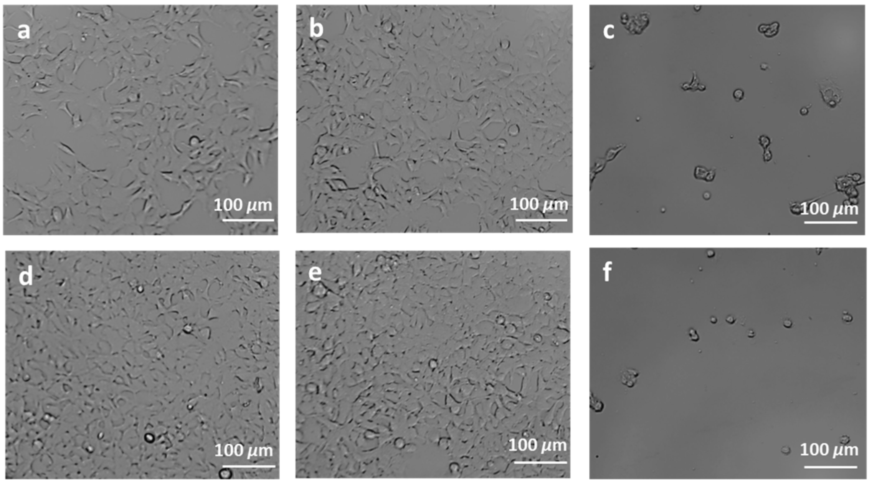

Figure S3. Representative morphologies of HEK-293 cells of each group on day 1 ((**a**) blank, (**b**) 20% SS/PEGDA, (**c**) positive control) and day 3 ((**d**) blank, (**e**) 20% SS/PEGDA, (**f**) positive control).

4. Standard calibration curve of berberine in diH_2O

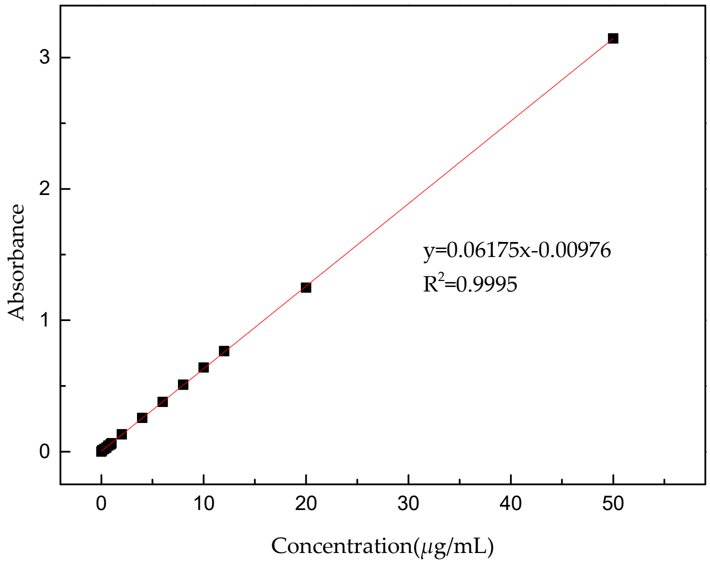

Figure S4. Standard calibration curve of berberine in diH_2O.

5. Standard calibration curve of berberine in PBS

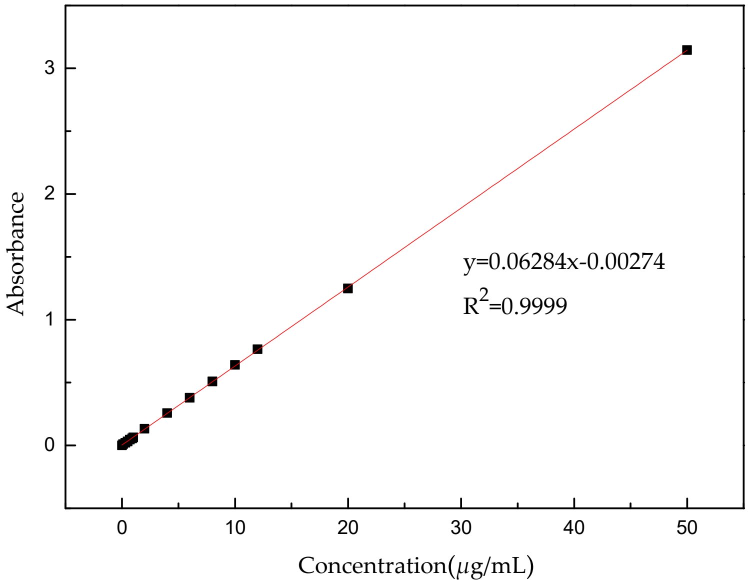

Figure S5. Standard calibration curve of berberine in PBS (pH = 7.4).
